# Molecular and Chemical Engineering of Bacteriophages for Potential Medical Applications

**DOI:** 10.1007/s00005-014-0305-y

**Published:** 2014-07-22

**Authors:** Katarzyna Hodyra, Krystyna Dąbrowska

**Affiliations:** Institute of Immunology and Experimental Therapy, Polish Academy of Sciences, R. Weigla 12, 53-114 Wrocław, Poland

**Keywords:** Bacteriophages, Phage display, Mutagenesis, Covalent immobilization, Vaccines, Carriers, Biosensors

## Abstract

Recent progress in molecular engineering has contributed to the great progress of medicine. However, there are still difficult problems constituting a challenge for molecular biology and biotechnology, e.g. new generation of anticancer agents, alternative biosensors or vaccines. As a biotechnological tool, bacteriophages (phages) offer a promising alternative to traditional approaches. They can be applied as anticancer agents, novel platforms in vaccine design, or as target carriers in drug discovery. Phages also offer solutions for modern cell imaging, biosensor construction or food pathogen detection. Here we present a review of bacteriophage research as a dynamically developing field with promising prospects for further development of medicine and biotechnology.

## Introduction

Recent progress in molecular engineering has contributed to the great progress of medicine. Biopharmaceutics such as hormones, interferons, interleukins, hematopoietic growth factors or therapeutic enzymes constitute a new class of drugs. They have found application in various diseases including anemia, leukemia, multiple sclerosis, diabetes and many others. The beginning of modern biotechnology dates back to 1982, when recombinant human insulin for the first time was introduced for treatment of diabetes. Later that success was continued with production of human growth hormone as the first fusion protein in 1985. Among promising molecular tools in biotechnology, molecular biology or medicine phages can be used. Phages have induced progress in such biotechnological branches as: biosensors, drug-carrying particles, cancer cell imaging agents, investigation of phage proteins’ structure and function, epitope mapping, studies of protein–protein interactions, determination of inhibitors’ and enzymes’ specificity, screening for receptor agonists and antagonists and finally for vaccine design, and anti-cancer research. These may contribute to the development of alternative methods in medicine, for examples in still serious medical problems like high risk of cancer or AIDS.

## Phage Display

Probably the first idea of phage application as a modern biotechnology tool was phage display. Phage display is a molecular technique that allows expression of exogenous proteins on a bacteriophage surface. The first report describing display of a foreign polypeptide on the surface of a bacteriophage particle comes from 1985 (Smith 1985). George P. Smith introduced a fragment of the *Eco*RI restrictase coding sequence into the middle section of gene *3* of the non-lytic filamentous phage f1. The new fusion protein P3-EcoRI did not destroy phage infectivity and the presented *Eco*RI retained antigenic properties of its native form. Filamentous phages that do not lyse infected bacteria during their propagation cycle have been most commonly used as phage display platforms (Huang et al. 2012a). Infection caused by these phages does not cause cell lysis, only “constant production”, which results in twice as slow growth of bacteria (Czaplicki 2005). However, T4 and T7 phages that represent lytic Caudovirales have also been used for phage display (Efimov et al. 1995). The lytic cycle results in the destruction of the infected cell after the phage penetrates into bacteria; the replication and expression of the bacterial genetic material changes in favor of the phage. After the assembly and maturation of virions, the cell wall is destroyed and viruses can infect other cells (Karam 1994).

Filamentous phage strains such as M13, fd or f1 (Smith and Petrenko 1997) are characterized by a flexible rod shape with a circular ssDNA genome. They infect *Escherichia coli* via the F pilus. The M13 capsid is built by 2,700 copies of major coat protein p8 and is capped by p3 (5 copies), p6 (5 copies), p7 (5 copies), and p9 (5 copies). All these coat proteins can be used as fusion targets for display, but p3 and p8 proteins are used most widely. Protein p8 is limited to displaying short peptide sequences, while p3 allows display of larger insertions (Pande et al. 2010).

The most popular way for expression of a foreign peptide or protein on the bacteriophage surface is the fusion proposed by Smith (1985). A gene encoding the foreign protein is fused to one of the M13-related viral coat protein genes. Filamentous phage expression is ideal for oligopeptides and small proteins (Bratkovič 2010); in the case of bigger proteins, this platform is insufficient. This problem has been solved by the introduction of phagemids as special helping display vectors (Bass et al. 1990). A phagemid is a plasmid with phage origin of replication and packing signal which can express a fusion protein but does not encode any viral structural or replication proteins. Fusion proteins are carried by phagemids while the majority of the genes required for the formation of phage particles are carried by helper phages that are co-infected together with phagemids into host bacteria (Sidhu 2001). Co-infection of the bacterial host cell by a phagemid and a phage produces hybrid virions displaying only a few copies of the fusion coat protein additionally to the majority of wild-type structural coat proteins (Pande et al. 2010). This system is called a “hybrid-phage system”, and has been created by Smith (Smith’s classification); this system is based on the arrangement of the coat proteins (McConnell et al. 1994). The authors introduced the terms 3, 33 or 3 + 3 (for p3-based display), 6, 66, or 6 + 6 (for p6-based display), and 8, 88, or 8 + 8 (for p8-based display) to differentiate possible protein arrangements (Smith and Petrenko 1997). Type 3, 6 and 8 are the simplest cases. A foreign protein is displayed on each copy of a phage protein in the capsid. Types 3 + 3, 6 + 6, and 8 + 8 of the system engage a combination of the phage and the phagemid, allowing combination of fusion proteins and wild proteins in the same capsid. Types 33, 66, 88 also allow one to combine fusion proteins and wild proteins in the same capsid, but they are expressed from the same phage genome (Bratkovič 2010).

Later, in a relatively short time, phage display was developed into a wide range of variations, employing differentiated phage strains and several technological approaches. Now, the multiplicity of phage display variations can by classified according to diverse aspects of these techniques (Table 1).

**Table 1:** Types of phage display

Types of proteins on phage capsid	Types of phage display				
	Only fusion proteins on the capsid		Both fusion and native coat proteins on the capsid		
Origin of fusion proteins displayed on a phage capsid	Permanent fusion constructed in the phage genome	Deletion of a non-essential phage coat protein and introduction of fusion protein • In vitro • In vivo	Fusion protein expressed from a plasmid and native coat protein expressed from phage genome	Fusion protein expressed from a phagemid and native coat protein expressed from helper phage genome	Fusion protein expressed from a phage genome and native coat protein expressed from the same phage genome
Examples	e.g. The most common type 3 (Smith 1985)	Incorporation of fusion proteins into phage capsid • In vitro (e.g. Sathaliyawala et al. 2006; Shivachandra et al. 2006; Jiang et al. 1997) • In vivo (e.g. Li et al. 2006, 2007; Oślizło et al. 2011; Ren and Black 1998)	e.g. Competitive phage display (Ceglarek et al. 2013)	e.g. Type 3 + 3 (Smith 1985)	e.g. Type 33 (Smith 1985)

Phage capsid in the phage display can contain only fusion protein or both fusion and native wild-type proteins. Phage display with fusion proteins can be grouped into two types: permanent fusion of phage and foreign genes in phage genome, which can be classified as type 3, 6 or 8 according to Smith’s ordination (description above). It can also be done by deletion of a non-essential phage coat protein followed by the introduction of a selected fusion to the phage capsid. This second type is based primarily on the T4 bacteriophage. The phage T4 capsid is built with two essential proteins, gp23* and gp24*, and two decorative proteins: Hoc (highly antigenic outer capsid protein) and Soc (small outer capsid protein). The in vivo display system allows for target fusion, which will be displayed on the phage surface to the capsid protein. Capsid proteins fused to foreign proteins or peptides are overexpressed in a bacterial system such as the *E. coli* system. During assembly, these fusions are incorporated into the phage capsid by simply mixing. Protein (Hoc-target or Soc-target) is built into hoc^−^ or soc^−^ phage by simply mixing. The phage strains used in the experiments with supplementary expression vectors had a deletion or a non-sense mutation in the gene, and thus no native gene products have been incorporated into its head during phage assembly. Since Hoc and Soc are not essential head proteins, these defects do not affect phage viability (Jiang et al. 1997; Oślizło et al. 2011; Ren et al. 1996; Ren and Black 1998). This system was used, e.g. to display full-length antigens from human immunodeficiency virus (HIV) (Li et al. 2007; Shivachandra et al. 2006). In vivo systems have been used on other phages such as λ or T7 phage (Castagnoli et al. 2001; Maruyama et al. 1994; Mikawa et al. 1996). One of the limitations of in vivo display is the fact that no control can be exerted on intracellular expression structure and assembly on foreign proteins. This problem is solved by the use of in vitro phage display. This system differs from that extended in vivo because of incorporation of target proteins to the capsid outer bacterial cell on mature bacteriophage particles. In vitro phage display has been reported in the first presentation of a 710 kDa anthrax toxin on bacteriophage T4 (Li et al. 2006).

Phage display with both fusion and native proteins, as its name suggests, engages two types of proteins at the same time: fusion proteins and wild-type protein. Fusion protein is made by fusion of foreign amino acid sequence to the endogenous amino acids of the coat protein. Fusion protein can be expressed from the plasmid (competitive phage display), phagemid (type 3 + 3) or phage genome (type 33).

## Phage Display Libraries

The most common applications of phage display constructs are random peptide libraries. A phage library is a collection of phages carrying on their surface foreign proteins or peptides encoded by DNA inserted into the phage genome (e.g. type 3 phage display) (Smith 1993). One of the most important functions of phage libraries is to deliver a diversified pool of elements. Each phage displays on its surface a single type of protein or peptide, but the whole library has a large number of viruses with many different proteins in total. Each viral vector is capable of infection, and therefore each phage selected from the phage library with the respective peptide can be separately replicated in bacteria. The method for the production of peptide libraries is based on Zoller and Smiths’s procedure (Zoller and Smith 1982, 1983); it allows a foreign nucleotide sequence to be introduced into the M13 vector, which causes formation of mutant clones capable of displaying a respective peptide. This method can be used to construct libraries of the size ranging 10^9^–10^11^ (Huang et al. 2012a). Scholle et al. (2005) created libraries with 100 % recombinant phages by the use of negative selection of *amber* stop codon at the 5′ end of the gene p3 in the filamentous phage.

One of the most popular applications of phage display libraries is selection for affinity domains of antibodies. In 1990, McCafferty and colleagues (1990) for the first time displayed complete V variable domains of antibodies on the surface of a filamentous phage. Thus, they initiated a new way of making antibodies demonstrated using polymerase chain reaction (PCR) reaction. Phage display antibody libraries are a combination of heavy-chain variable domain (VH) with light-chain variable domain (VL) which are presented on a phage surface (Pini and Bracci 2000). These two domains together form the variable domain (V domain) which is responsible for antigen binding and unique specificity. The traditional molecular method for producing antibody fragments uses the phagemid vector including the V domain from lymphocytes which is amplified by PCR (Table 2).

**Table 2:** Types of antibody phage display

Types of antibody phage display			
Immunization of animals with target molecule	Yes (post-immunization libraries)	No (single-pot libraries)	
V-gene construction	In vivo IgG mRNA from B cells	In vivo (naïve libraries) IgM mRNA from B cells	In vitro (semi-synthetic libraries) Random sequence of antibody coding genes mRNA from pre-B cells

There are two categories of antibody libraries: post-immunization libraries and single-pot libraries. The first type contains the immunoglobulin G (IgG) sequence derived from the spleen B cells of immunized animals. V genes are isolated and assembled into functional antibody fragment (mostly scFv or Fab) and inserted into phage library vectors. Post-immunization libraries have high affinity to an antigen; it is however necessary to construct a new library for every antigen. Single-pot libraries use B cells from unimmunized donors. Two types of single-pot library have been developed: naïve and synthetic. Naïve libraries are constructed using V genes sequences that have undergone some natural rearrangement, for example those derived from IgM mRNA. Synthetic libraries are built in vitro on rearranged antibody gene segments with some additional sequences; therefore, good knowledge of the complementary determining region (CDR) sequences, which are critical for antigen binding, is necessary (Smith et al. 2005; Willats 2002).

## Phage Display Applications

Phage display can be used in a variety of applications as presented in exemplary use in experimental animal or in vivo models, e.g. for epitope mapping (Fack et al. 1997), studies of protein–protein interactions (Sidhu et al. 2013), to determine specificity of inhibitors and enzymes (Diamod 2007; Hawinkels et al. 2007), in screening for receptor agonists and antagonists (Lee et al. 2001), or in vaccine design (De Berardinis and Haigwood 2004; Ren et al. 1996; Ren and Black 1998; Sathaliyawala et al. 2006). Due to the wide range of possibilities offered by phage display, this method is postulated as a tool for looking of solution in many medical problems.

The most popular one is screening for anticancer peptides or proteins. There are many publications describing peptides’ selection by phage display. These peptides are able to influence angiogenesis and tumor cell growth (Cieslewicz et al. 2013; Lu et al. 2012; Mai et al. 2009). One of the best characterized and important factors causing angiogenesis is vascular endothelial growth factor (VEGF); moreover, this factor is responsible for tumor growth stimulation. It is the key mediator of angiogenesis (the formation of new blood vessels), and binds two types of VEGF receptors (VEGFR1 and VEGFR2) (Carmeliet 2005). It has been demonstrated that VEGF is responsible for stimulation and cancer metastasis, and therefore is a frequent therapeutic target. The VEGF family and its receptor system has been shown to be the fundamental regulator in the cell signaling of angiogenesis (Lu et al. 2003). The phage display method allows one to select peptides that are capable of inhibition of tumor growth (Borysowski and Górski 2004). From a large human naïve antibody library four fully human anti-KDR (KDR, kinase insert domain-containing receptor or VEGFR2) antibodies were identified and it was demonstrated that these antibodies were able to block KDR/VEGF interaction and neutralize VEGF-induced angiogenic activity (Lu et al. 2002). Other studies described ^90^Y-labeled nanoparticles targeted to the vasculature with anti-VEGFR antibodies for anti-tumor therapy (Li et al. 2004).

Fibroblast growth factor 8b (FGF8b) is the major isoform of FGF8 expressed in prostate cancer and it correlates with the stage and grade of the disease. Using the phage display method 12 specific FGF8b-binding phage clones were constructed and isolated by screening a phage display heptapeptide library with FGF8b, which was named as P12. Studies suggested that P12 may have a greater potential to interrupt FGF8b binding to receptors than others, which were identified by phage display libraries. Functional analysis indicated that synthetic P12 peptides mediate significant inhibition of FGF8b-induced cell proliferation and blockade of the activation of Erk1/2 and Akt cascades in both prostate cancer cells and vascular endothelial cells (Wang et al. 2013).

Cysteine-rich protein 61 (CCN1/Cyr61) has been used as an important mediator in proliferation and metastasis of breast cancer; blockage of Cyr61 might be a potent target for breast cancer treatment. Using the phage display system developed antibody denoted as 093G9, an inhibitory effect on breast human cell line proliferation and migration was induced. Additionally, 093G9 also showed significant efficacy on suppressing primary tumor growth and spontaneous lymph node metastasis in vivo in a mouse model (Lin et al. 2012).

High exposure of selected antigens on the phage display platform and the relative ease of their production make phage display a promising tool for constructing vaccines. This includes the challenge of developing an effective vaccine for HIV (Burton et al. 2004; Esparza 2005). Bacteriophage T4 has been proposed as a recombinant platform that allows construction of multicomponent vaccine boosting humoral and cellular responses (Ren et al. 1996; Ren and Black 1998; Sathaliyawala et al. 2006). The head of T4 bacteriophage is an icosahedron with one portal vertex (120 × 86 nm) to which the phage tail is attached (Fokine et al. 2004; Rao and Black 2010). The icosahedral caps are formed by hexamers of gp23* and pentamers of gp24*, which are essential proteins. Hoc and Soc are two nonessential proteins (Leiman et al. 2003). The nonessential proteins have a number of features that recommend them as alternative vehicles for protein display. Ren fused the V3 loop domain of gp120 (HIV-1 envelope glycoprotein) to Soc protein. Soc-V3 fusion was expressed in the *E. coli* system, and then bound in vitro to the phage. Soc-V3 displaying phage were highly antigenic in mice and produced antibodies reactive with native gp120 (Ren et al. 1996). Further, a Hoc-based in vivo assembly system allowed display of HIV antigens p24-gag, Nef and gp41 C-peptide trimer, which represent differentiated structures and biological function. P24 displayed as a Hoc-fusion was highly immunogenic in mice in the absence of any external adjuvant, eliciting strong p24-specific antibodies, as well as Th1 and Th2 cellular responses with a bias toward the Th2 response. The phage T4 system, which was used in these experiments, increases the range of immune responses; therefore, it was proposed for HIV vaccine development (Ren et al. 1996; Ren and Black 1998; Sathaliyawala et al. 2006).

Phage display can also be used to design new antibacterial peptides. This may be useful when phages specific to difficult pathogens are not available, which has been shown in the case of phage specificity to *Staphylococcus aureus* and *Helicobacter pylori*. *S. aureus* cause many diseases by producing toxins, such as pneumonia, endocarditis, meningitis, septicemia, and toxic shock syndrome (Lowy 1998). A large percentage of *S. aureus* infections are caused by MRSA (Methicillin-resistant *S. aureus*). Development of resistance to available antibiotics has become a serious problem in treatment of *S. aureus* infections (Lowy 2003). There is therefore an imperative need to find new types of antibacterial agents. *H. pylori* is a Gram-negative bacterium which produces the virulence factor urease. This allows survival of the bacteria in the acidic environment of the stomach. *H. pylori* causes many serious diseases including duodenal ulcers and stomach cancer. Antibiotic therapy has significant limitations, such as the high cost and the emergence of antibiotic-resistant strains, generating the need for new ways of treatment. For selection of new specific peptides capable of binding difficult bacterial pathogens, phage display library screening can be utilized (Ardekani et al. 2013).

Phage display libraries are used for identification of specific peptides and antibodies against pathogen targets. Synthesis of virulence factors produced by *S. aureus* is regulated by a quorum-sensing mechanism. *S. aureus* secretes a protein termed RNAIII activating protein (RAP) which autoinduces toxin. Young and colleagues showed that mice which have been vaccinated with selected RAP were protected from *S. aureus* infection, which suggested that RAP is a useful target for selecting potential therapeutic molecules to inhibit this pathogen (Yang et al. 2003). Peptides recognizing staphylococcal enterotoxin B (SEB) selected from the M13 phage library were applied to attenuate this bacterium. SEB is a pyrogenic toxin responsible for staphylococcal food poisoning in humans and has been an attractive choice as a biological aerosol weapon due to its inherent stability and high intoxication effect (Ler et al. 2006). Three peptides of high affinity to SEB (WRPLTPESPPA, MNLHDYHRLFWY, QHPQINQTLYRM) were highly active against *Staphylococcus* (Soykut et al. 2008).

Phage display technology was used for identification of peptides able to bind specifically and to inhibit *H. pylori* urease such as 24-mer TFLPQPRCSALLRYLSEDGVIVPS and 6-mer YDFYWW that can inhibit the activity of urease purified from *H. pylori* (Houimel et al. 1999). In another approach, an antibody display was developed for a single-variable domain of heavy chain antibody against recombinant UreC. The isolated UreC nanobody can specifically detect and bind to UreC and inhibit urease activity (nanobodies are isolated variable domains of heavy chain antibodies). This nanobody could be a novel class of treatment measure against *H. pylori* infection (Ardekani et al. 2013). The identification of inhibitory peptides and nanobodies specific for *H. pylori* urease may open a new approach for the development of therapeutic drugs.

Recently, phage display was applied in purification of bacteriophages. New methods combining phage display and chromatography give good results in separating T4 bacteriophages from bacterial proteins, DNA, lipopolysaccharides and even from other phages (Ceglarek et al. 2013; Oślizło et al. 2011). It is a very useful technique for purification of phages compared to other methods, e.g. gradient centrifugation in cesium or saccharose, which are more time-consuming operations. Moreover, because of this method phages are highly purified. There are two methods, one based on competitive phage display, in which bacterial cells produce both wild-type proteins (expressed from the phage genome) and the protein fusions with affinity tags (expressed from expression vectors). These tags are glutathione S-transferase (GST) or six histidine (His-tag); phage proteins fused to the tags are incorporated into the phage capsid during assembly.

## Random Mutagenesis of Bacteriophages

Random mutagenesis is the process of introducing changes inside bacteriophage DNA without pre-designing these changes. Random mutagenesis can be spontaneous or induced. Spontaneous mutagenesis results from errors in DNA replication; this kind of mutant occurs frequently in the environment. Random mutations can also be inducted by many physical or chemical factors. Physical factors causing changes in DNA are ionization, UV radiation and temperature. Acridine dyes, nitrogenous base analogs, nitric acid, some hydrocarbons and others are chemical inductors of mutations. Mutations induced by physical or chemical mutagens are a very popular approach in research methodologies. They make it possible to examine functions of genes based on changes in phenotype characteristics.

At the molecular level, random mutagenesis techniques base on typical kinds of point mutations, including transitions, transversions, frameshift mutations and deletions. The principal strategy of random mutagenesis is formation of random mutations by chemical or physical mutagens, and selection of mutants with altered properties. This technique does not require detailed knowledge of the phage. The main point is the modification of phage phenotype and the assumption that mutation in the genome is responsible for the new virus properties. Random mutagenesis is more of a random modification at the molecular level than genetic engineering; however, when well planned, it allows for the selection of a phage even with very sophisticated properties. A good example is provided by the studies of Merril et al. (1996). They isolated λ and P22 phage mutants which can circulate longer in mice in comparison to wild-type phages, which was related to one point mutation located in a major head protein coding gene. Another example of the application of random mutagenesis is identification and selection of their most able T4 lysozyme. Two variants were found, which exhibited increased reversible melting temperatures with respect to the wild-type protein. The results also illustrate the power of random mutagenesis in obtaining variants with a desired phenotype (Pjura and Matthews 1993). UV irradiation is one of the most popular physically induced methods of mutagenesis. T4 phage can be inactivated by UV light at a wavelength of 253.7 mµ. Mutation in a single gene ν that controls the repair process by photoreaction causes increased UV sensitivity in T4 phage (Harm 1963). Hall and colleagues have applied bacteriophages in studies of coevolutionary dynamism of phages and their hosts. These authors examined interaction between bacteria and a phage by the use of a parasitic phage with random mutations in its tail fiber. They demonstrated that random mutations could be a very useful tool for investigation of a phage influence on the genetic divergence of bacterial hosts. One of the most interesting conclusions was that bacteriophages were more likely to emerge through long-term coevolution with their hosts than through spontaneous adaption to a single novel host (Hall et al. 2011a, b).

Site-directed mutagenesis is a phage modification technique leading to introduction of designed changes into a specific site in the genome. It involves specially designed synthetic oligonucleotides, i.e. primers for PCR. Mutagenic primers are complementary to the part of the engineered DNA template, but they also contain an internal mismatch which codes for a designed change of the selected genes. Specified changes in nucleotide sequences of a gene lead to modification of amino acid sequences in the protein often enabling introduction or removal of a considerable number of amino acids.

Thus site-directed mutagenesis allows studies of molecular determinants of protein structure and function (Switala-Jelen et al. 2002).

A molecular tool that allows for the introduction of site-directed mutagenesis is the bacteriophage insertion-substitution (I/S) vector system. This system was first derived by Selick et al. (1988) for T4 bacteriophage. This method enables the transmissions of in vitro-generated mutations from a plasmid into the phage gene. The mutation insert is first constructed as a cloned insert within the I/S plasmid. Bacteria containing this plasmid are then infected with bacteriophage that carries *amber* mutations in selected genes. The plasmid integrates the mutation-containing sequence into the phage genome by homologous recombination. Integrant phage mutants are selected by comparison of their growth on the *amber*-suppressing and non-suppressing bacterial strains.

This technique allows for introduction of almost any changes in the genes. Therefore, site-directed mutagenesis is a very popular technique that has been used for studying gene function and protein structure/function. Site-directed mutagenesis has helped in understanding the function and structures of some important gene products such as gene 32 from T4 phage (Shamoo et al. 1989) or the role of D-loop using ATP binding cassette proteins from T4 phage and gp47 (De la Rosa and Nelson 2011). Moreover, it is widely used for engineering disulfide bonds in the case of T4 lysozyme and improves its stability (Perry and Wetzel 1984).

Site-directed mutagenesis is in fact a molecular tool for constructing recombinant phage libraries which display foreign peptides on surfaces (Huang et al. 2012b). It is used for constructing libraries of protein variants in M13 bacteriophage (Fellouse et al. 2007; Huang et al. 2012b). This powerful technique allows us to obtain phage display libraries that comprise a vast number of mutants, as it has been observed that the size of a phage library is closely correlated with the affinity of the isolated mutants (Ling 2003).

Site-directed mutagenesis was recently studied by Yoichi et al. (2005). They modified phage lytic spectrum by changes in tail fiber protein gp38 of T2 bacteriophage. In that research, homologous recombination between T2 phage genome and a plasmid encoding the region around *genes 37*-*38* from PP01 phage was used. Authors obtained recombinant T2ppD1phage derived from T2 which carried gp37 and gp38 from PP01 phage. Insertion of foreign gp37 and gp38 into T2 phage conferred infectivity of the heterogeneous host *E. coli* O157:H7 which was not sensitive to the wild-type of T2. *E. coli* K12 strains which was the original host of T2, could not be infected by recombinant T2ppD1 phage. Site-directed mutagenesis could be employed for customizing of phage host range according to specific needs. This may have practical significance for phage therapy and identification of bacteria (Yoichi et al. 2005).

Bacteriophage recombineering of electroporated DNA (BRED) is the most powerful technique allowing for phage mutant creation. It has been mainly applied on temperate phages but developments for lytic phages are also emerging. This approach was first used in mycobacteriophages. BRED can be used for the construction of unmarked detection in essential as well as nonessential genes. It can take advantage of creation in-frame internal deletions, point mutations, nonsense mutations and additions of gene tags. BRED strategy allows for co-electroporation of phage DNA temple and targeting substrate to recombineering *Mycobacterium smegmatis* cells. This strategy offers a very high effectiveness of mutant construction; as reported by the authors more than 10 % of finally recovered plaques contained the desired mutant (Marinelli et al. 2008; van Kessel and Hatfull 2008). Recombinieering phages technique has been applied also for λ phage (Oppenheim et al. 2004). There are many potential possibilities offered by BRED technology, mostly for investigations of bacteriophage genetics. Phage genomes can by modified with genes of unknown function which may allow for the function identification.

New concept for using phages as detection tools are fluorescent and luminescent-labeled phages. Bacterial lucyferase gene (*lux*) was transferred to a phage genome for first time by Ulitzur and Kuhn (1987). The gene was cloned into the bacteriophage λ genome. Reporter phages with lucyferase gene were demonstrated as creative tools for rapid detection of bacterial host cells following phage infection. The minimum number of cells which can be detected did not exceed ten for *E. coli* (Ulitzur and Kuhn 1987), one hundred for *Salmonella typhimurium* (Stewart et al. 1989), or ten for enterobacterial cells (Kodicara et al. 1991). Loessner et al. (1996) developed new recombinant phage against *Listeria*: A511::*luxAB* by introduction of the *luxAB* genes into the gene coding major capsid phage protein. After infection they observed a high level of luciferase expression in bacterial cells (Loessner et al. 1996). These bacteria are food borne pathogens which gives a high practical potential to this new method of identification.

Other approach that employs light for phage detection is that based on green fluorescent protein (GFP) introduced to the phage capsid. This technique combines mutagenesis and phage display method. Small outer capsid (SOC) protein of PP01 phage was used as a platform to present a marker protein GFP. Fusion of GFP to SOC did not change host range of the phage, interestingly, binding of the recombinant phage to bacterial cells enhanced. Adsorption of the GFP-labeled PP01phages to the *E. coli* cell surface enabled visualization of cells under a fluorescence microscope. The fluorescence of GFP within infected bacteria enables highly sensitive detection (Oda et al. 2004). Kaźmierczak et al. (2014) proposed T4 phage as a new tool for molecular imaging of bacteriophages in living systems. They used T4 phage mutant HAP (T4 without decorative Hoc protein). GFP was fused to the N-terminus of Hoc by in vivo phage display. Fluorescent phages were positively assessed as regards their applicability for detection inside living mammalian cells (by phagocytosis) and tissues (filtering and retention by lymph nodes and spleen) (Kaźmierczak et al. 2014).

Another application of site-directed mutagenesis is using this method for affinity maturation of antibodies or for epitope mapping. These approaches usually combine construction of phage-displayed antibody libraries with mutagenesis. Parent antibody sequence is subjected to mutagenesis which is followed by optimized selection of affinity-improved variants. These variants are selected against a relevant target. Combination of phage display and site-directed mutagenesis is usually applied to understand the structure, function and interactions between antibodies and antigens (Infante et al. 2014; Lamdan et al. 2013).

## Chemical Modification of Bacteriophage Particles

Most chemical modifications of bacteriophages are based on conjugation of some prosthetic groups to the phage surface. Various chemical compounds can be attached to the surface protein in a specific reaction at the appropriate temperature, incubation time and favorable pH. Conditions of the incubation reactions are relatively non-aggressive, so the phage does not lose its biological properties. By this reaction, such reactive groups can be attached as amino groups of lysine residues, carboxylic acid groups of aspartic acid, glutamic acid residues, the phenol group of tyrosine residues, some ester bond linkers and a chemical monolayer. To these linkers can be further attached to folic acid, some fluorescent markers, or even various antibiotics. Moreover, chemical conjugations allow one to attach polyethylene glycol particles directly to a phage.

Li and colleagues’ data showed a potential application of the M13 phage in cell imaging. For dual modification of M13 they chose the most reactive group, tyrosine, as the site where fluorescent dye and folic acid were attached. Folic acid is one of the most common ligands for cancer cell targeting. Dual-modified M13 showed very good binding to HeLa contaminant of KB cells, thus demonstrating the potential of chemically modified M13 in bio-imaging and drug delivery (Li et al. 2010).

Chemical modification of phages allows them to be used in targeted drug therapy. A very interesting research paper by Yacoby et al. (2007) presented bacteriophage M13 as a platform of targeted drug carriers for the eradication of pathogenic bacteria.

The same phage was used for the conjugation of antibiotic and IgG antibodies. IgG attached on each P3 protein allowed for recognition of the target and P8 coat protein with a conjugated drug designed to kill bacteria. A schematic representation of antibacterial-targeted drug carrying bacteriophages can be summarized in three steps: (1) preparing a pro-drug; (2) conjugating the pro-drug to the phage; (3) drug-carrying phage binding to the target bacteria and drug release.

This research offered a new approach to selective drugs of a targeted specificity by the attached elements, which may allow the reintroduction of nonspecific drugs that have thus far been excluded from antibacterial use because of toxicity or low selectivity. Drug-currying phage has high selectivity against bacteria; in general use, it may help to combat emerging bacterial antibiotic resistance (Yacoby et al. 2006).

PEGylation is a very noteworthy type of chemical modification. It allows for phage structural protein conjugation with polyethylene glycol (PEG). After PEGylation bacteriophages do not change their specificity to bacteria. In vivo and in vitro experiments with mice showed that PEGylation of bacteriophages is efficient to delay virus clearance and achieve longer circulation in non-immunized mice. Additionally, PEGylation can reduce the cellular immune response such as antigen-specific T cell proliferation (Kim et al. 2008). This chemical modification of bacteriophages may have great significance for phage therapy against antibiotic-resistant bacteria. Nowadays, antibiotic resistant bacteria represent an important medical problem. An increasing number of drug resistant-bacterial infections are very difficult to treat (Górski et al. 2009). Bacteriophages are an alternative to antibiotics. They can be used as a useful tool against pathogenic bacteria. Special properties of PEGylated bacteriophages such as long persistence in the organism together with drug-targeting bacteriophages may contribute to the development of phage therapy.

Bacteriophages are viruses which can recognize and bind specific bacterial receptors and create covalent bonds between phage particles and gold surfaces. This method is used for constructing biosensors by phage covalent immobilization. Detection of *E. coli* K12 can be done by covalent immobilization of T4 bacteriophages onto gold surfaces using a self-assembled monolayer of dithiobis(succinimidyl propionate) (DTSP) (Arya et al. 2011). *E. coli* is a natural inhabitant of the intestinal tracts of humans and warm-blooded animals. Although some pathogenic strains of *E. coli* cause diarrhea, bloody feces, kidney failure, hemolytic uremic syndrome and even death, it is a common food-borne pathogen (Ho et al. 2004). Bacteriophage T4 can recognize and bind to specific receptors on the *E. coli* host using T4 tail spike proteins (Karam 1994; Kutter and Sulakvelidze 2005). A bioassay platform utilizing T4 bacteriophages via a specific receptor has been developed for the detection of *E. coli* K12 bacteria.

Petrenko (2008) has pointed out the potential of bacteriophages in creation of bioselective materials and eventually in constructing phage-derived analytical platforms. Phages can be used as a recognition element in biosensors by using physical adsorption to immobilize phage on the sensor surface. Filamentous phages are most commonly used in biosensor construction. This phages characterized by simple composition. The major desired characteristics of the biosensors are their sensitivity, selectivity, robustness and prompt performance. Phage layers bind biological agents with high affinity and specificity and generate detectable signals in analytical platforms, for instance, it can be used for detection of *Bacillus anthracis* spores and *S. typhimurium* cells (Petrenko 2008). Biosensors may solve the problem of detection of many pathogenic and food borne bacteria or their when their concentration is low (for standard detection methods) but still harmful for the consumers’ health.

## Conclusions

Nowadays medicine is still intensively searching for new and imaginative solutions of difficult problems. These problems are for example, new generations of anticancer agents that would allow for replacement of traditional ones, new types of biosensors that may help in easy detection of still dangerous bacteria, or vaccine development since we still face the threat of HIV and other difficult pathogens. Phages offer solutions for many of those challenges. They are a very useful tool in medicine and biotechnology, showing encouraging results. Phage can be applied as anticancer agents, novel platforms in vaccine design, or as target carriers in drug discovery. They offer solutions for modern cell imaging, biosensor construction or food pathogen detection. Bacteriophage research is a dynamically developing field with promising prospects for further development of medicine and biotechnology.

## References

[CR1] Ardekani LS, Gargari SL, Rasooli I (2013). A novel nanobody against urease activity of *Helicobacter pylori*. Int J Infect Dis.

[CR2] Arya SK, Singh A, Naidoo R (2011). Chemically immobilized T4-bacteriophage for specific *Escherichia coli* detection using surface plasmon resonance. Analyst.

[CR3] Bass S, Greene R, Wells JA (1990). Hormone phage: an enrichment method for variant proteins with altered binding properties. Proteins.

[CR4] Borysowski J, Górski A (2004). Phage-display technology and its application to experimental oncological therapy (in Polish). Postepy Hig Med Dosw.

[CR5] Bratkovič T (2010). Progress in phage display: evolution of the technique and its application. Cell Mol Life Sci.

[CR6] Burton DR, Desrosiers RC, Doms RW (2004). HIV vaccine design and the neutralizing antibody problem. Nat Immunol.

[CR7] Carmeliet P (2005). VEGF as a key mediator of angiogenesis in cancer. Oncology.

[CR8] Castagnoli L, Zucconi A, Quondam M (2001). Alternative bacteriophage display systems. Comb Chem High Throughput Screen.

[CR9] Ceglarek I, Piotrowicz A, Lecion D (2013). A novel approach for separating bacteriophages from other bacteriophages using affinity chromatography and phage display. Sci Rep.

[CR10] Cieslewicz M, Tang J, Yu JL (2013). Targeted delivery of proapoptotic peptides to tumor-associated macrophages improves survival. Proc Natl Acad Sci USA.

[CR11] Czaplicki D (2005). Phage-displayed peptide libraries and application in cancer research (in Polish). Postepy Biologii Komorki.

[CR12] De Berardinis P, Haigwood NL (2004). New recombinant vaccines based on the use of prokaryotic antigen-display systems. Expert Rev Vaccines.

[CR13] De la Rosa MB, Nelson SW (2011). An interaction between the Walker A and D-loop motifs is critical to ATP hydrolysis and cooperativity in bacteriophage T4 Rad50. J Biol Chem.

[CR14] Diamod SL (2007). Method of mapping protease specificity. Curr Opin Chem Biol.

[CR15] Efimov VP, Nepluev IV, Mesyanzhinov VV (1995). Bacteriophage T4 as a surface display vector. Virus Genes.

[CR16] Esparza J (2005). The global HIV vaccine enterprise. Int Microbiol.

[CR17] Fack F, Hügle-Dörr B, Song D (1997). Epitope mapping by phage display: random versus gene-fragment libraries. J Immunol Methods.

[CR18] Fellouse FA, Esaki K, Birtalan S (2007). High-throughput generation of synthetic antibodies from highly functional minimalist phage-displayed libraries. J Mol Biol.

[CR19] Fokine A, Chipman PR, Leiman PG (2004). Molecular architecture of the prolate head of bacteriophage T4. Proc Nalt Sci USA.

[CR20] Górski A, Miedzybrodzki R, Borysowski J (2009). Bacteriophage therapy for the treatment of infections. Curr Opin Investig Drugs.

[CR21] Hall AR, Pauline D, Scanlan PD (2011). Bacteria-phage coevolution and the emergence of generalist pathogens. Am Nat.

[CR22] Hall AR, Scanlan PD, Morgan AD (2011). Host-parasite coevolutionary arms races give way to fluctuating selection. Ecol Lett.

[CR23] Harm W (1963). Mutants of phage T4 with increased sensitivity to ultraviolet. Virology.

[CR24] Hawinkels LJ, van Rossenberg SM, de Jonge-Muller ES (2007). Efficient degradation-aided selection of protease inhibitors by phage display. Biochem Biophys Res Commun.

[CR25] Ho JA, Hsu HW, Huang MR (2004). Liposome-based microcapillary immunosensor for detection of *Escherichia coli* O157:H7. Anal Biochem.

[CR26] Houimel M, Mach JP, Corthesy-Theulaz I (1999). New inhibitors of *Helicobacter pylori* urease holoenzyme selected form phage-displayed peptide libraries. Eur J Biochem.

[CR27] Huang JX, Bishop-Hurley SL, Cooper MA (2012). Development of anti-infectives using phage display: biological agents against bacteria, viruses, and parasites. Antimicrobial Agents Chemother.

[CR28] Huang R, Fang P, Kay BK (2012). Improvements to the Kunkel mutagenesis protocol for constructing primary and secondary phage-display libraries. Methods.

[CR29] Infante YC, Pupo A, Rojas G (2014). A combinatorial mutagenesis approach for functional epitope mapping on phage-displayed target antigen: application to antibodies against epidermal growth factor. MAbs.

[CR30] Jiang J, Abu-Shilbayeh L, Rao VB (1997). Display of a PorA peptide from *Neisseria meningitides* on the bacteriophage T4 capsid surface. Infect Immun.

[CR31] Karam JD (1994). Molecular biology of bacteriophage T4.

[CR32] Kaźmierczak Z, Piotrowicz A, Owczarek B (2014). Molecular imaging of T4 phage in mammalian tissues and cells. Bacteriophage.

[CR33] Kim KP, Cha JD, Jang EH (2008). PEGylation of bacteriophages increases blood circulation time and reduces T-helper type 1 immune response. Microb Biotechnol.

[CR34] Kodicara CP, Crew HH, Stewart GS (1991). Near on-line detection of enteric bacteria using lux recombinant bacteriophage. FEMS Microbiol Lett.

[CR35] Kutter E, Sulakvelidze A (2005). Bacteriophages: biology and application.

[CR36] Lamdan H, Gavilondo JV, Muñoz Y (2013). Affinity maturation and fine functional mapping of an antibody fragment against a novel neutralizing epitope on human vascular endothelial growth factor. Mol BioSyst.

[CR37] Lee SC, Ibdah R, Van Valkenburgh C (2001). Phage display mutagenesis of the chimeric dual cytokine receptor agonist myelopoietin. Leukemia.

[CR38] Leiman PG, Kanamaru S, Mesyanzhinov V (2003). Structure and morphogenesis of bacteriophage T4. Cell Mol Life Sci.

[CR39] Ler SG, Lee FK, Gopalakrishnakone P (2006). Trends in detection of warfare agents: detection methods for ricin, staphylococcal enterotoxin B and T-2 toxin. J Chromatogr A.

[CR40] Li L, Wartchow CA, Danthi SN, Shen Z (2004). A novel antiangiogenesis therapy using an integrin antagonist or anti-Flk-1 antibody coated 90Y-labeled nanoparticles. Int J Radiat Oncol Biol Phys.

[CR41] Li Q, Shivachandra SB, Leppla SH, Rao VB (2006). Bacteriophage T4 capsid A unique platform for efficient surface assembly of macromolecular complexes. J Mol Biol.

[CR42] Li Q, Shivachandra SB, Zhang Z (2007). Assembly of the small outer capsid protein, Soc, on bacteriophage T4: a novel system for high density display of multiple large anthrax toxins and foreign proteins on phage capsid. J Mol Biol.

[CR43] Li K, Chen Y, Li S, Nguyen HG, Niu Z (2010). Chemical modification of M13 bacteriophage and its application in cancer cell imaging. Bioconjug Chem.

[CR44] Lin J, Huo R, Wang L (2012). A novel anti-Cyr61 antibody inhibits breast cancer growth and metastasis in vivo. Cancer Immunol Immunother.

[CR45] Ling MM (2003). Large antibody display libraries for isolation of high-affinity antibodies. Comb Chem High Throughput Screen.

[CR46] Loessner MJ, Rees CE, Stewart GS (1996). Construction of luciferase reporter bacteriophage A511::luxAB for rapid and sensitive detection of viable Listeria cells. Appl Environ Microbiol.

[CR47] Lowy FD (1998). *Sathyloccocusaureus* infection. N Engl J Med.

[CR48] Lowy FD (2003). Antimicrobial resistance: the example of *Staphylococcus aureus*. J Clin Invest.

[CR49] Lu D, Jimenez X, Zhang H (2002). Selection of high affinity human neutralizing antibodies to VEGFR2 from a large antibody phage display library for antiangiogenesis therapy. Int J Cancer.

[CR50] Lu D, Shen J, Vil MD (2003). Tailoring in vitro selection for a picomolar affinity human antibody directed against vascular endothelial growth factor receptor 2 for enhanced neutralizing activity. J Biol Chem.

[CR51] Lu G, Zheng M, Zhu Y (2012). Selection of peptide inhibitor to matrix metalloproteinase-2 using phage display and its effects on pancreatic cancer cell lines PANC-1 and CFPAC-1. Int J Biol Sci.

[CR52] Mai J, Song S, Rui M (2009). A synthetic peptide mediated active targeting of cisplatin liposomes to Tie2 expressing cells. J Control Release.

[CR53] Marinelli LJ, Piuri M, Swigoňová Z (2008). BRED: a simple and powerful tool for constructing mutant and recombinant bacteriophage genomes. PLoS ONE.

[CR54] Maruyama IN, Maruyama HI, Brenner S (1994). Lambda foo: a lambda phage vector for the expression of foreign proteins. Proc Natl Acad Sci USA.

[CR55] McCafferty J, Griffiths AD, Winter G (1990). Phage antibodies: filamentous phage displaying antibody variable domains. Nature.

[CR56] McConnell SJ, Kendall ML, Reilly TM (1994). Constrained peptide libraries as a tool for finding mimotopes. Gene.

[CR57] Merril RM, Biswas B, Carlton R (1996). Long-circulating bacteriophage as antibacterial agents. Proc Natl Acad Sci USA.

[CR58] Mikawa YG, Maruyama IN, Brenner S (1996). Surface display of proteins on bacteriophage lambda heads. J Mol Biol.

[CR59] Oda M, Morita M, Unno H (2004). Rapid detection of *Escherichia coli* O157:H7 by using green fluorescent protein-labeled PP01 bacteriophage. Appl Environ Microbiol.

[CR60] Oppenheim AB, Rattray AJ, Bubunenko M (2004). In vivo recombineering of bacteriophage lambda by PCR fragments and singlestrand oligonucleotides. Virology.

[CR61] Oślizło A, Miernikiewicz P, Piotrowicz A (2011). Purification of phage display-modified bacteriophage T4 by affinity chromatography. BMC Biotechnol.

[CR62] Pande J, Szewczyk MM, Grover AK (2010). Phage display: concept, innovations, applications and future. Biotechnol Adv.

[CR63] Perry LJ, Wetzel R (1984). Disulfide bond engineered into T4 lysozyme: stabilization of the protein toward thermal inactivation. Science.

[CR64] Petrenko VA (2008). Landscape phage as a molecular recognition interface for detection devices. Microelectronics J.

[CR65] Pini A, Bracci L (2000). Phage display of antibody fragments. Curr Protein Pept Sci.

[CR66] Pjura P, Matthews BW (1993). Structures of randomly generated mutants of T4 lysozyme show that protein stability can be enhanced by relaxation of strain and by improved hydrogen bonding via bound solvent. Protein Sci.

[CR67] Rao VB, Black LW (2010). Structure and assembly of bacteriophage T4 head. Virol J.

[CR68] Ren Z, Black LW (1998). Phage T4 SOC and HOC display of biologically active, full-length proteins on the viral capsid. Gene.

[CR69] Ren ZJ, Lewis GK, Wingfield PT (1996). Phage display of intact domains at high copy number: a system based on SOC, the small outer capsid protein of bacteriophage T4. Protein Sci.

[CR70] Sathaliyawala T, Rao M, Maclean DM (2006). Assembly of human immunodeficiency virus (HIV) antigens on bacteriophage T4: a novel in vitro approach to construct multicomponent HIV vaccines. J Virol.

[CR71] Scholle MD, Kehoe JW, Kay BK (2005). Efficient construction of a large collection of phage-displayed combinatorial peptide libraries. Comb Chem High Throughput Screen.

[CR72] Selick HE, Kreuzer KN, Alberts BM (1988). The bacteriophage T4 insertion/substitution vector system. A method for introducing site-specific mutations into the virus chromosome. J Biol Chem.

[CR73] Shamoo Y, Ghosaini LR, Keating KM (1989). Site-specific mutagenesis of T4 gene 32: the role of tyrosine residues in protein-nucleic acid interactions. Biochemistry.

[CR74] Shivachandra SB, Rao M, Janosi L (2006). In vitro binding of anthrax protective antigen on bacteriophage T4 capsid surface through Hoc-capsid interactions: a strategy for efficient display of large full-length proteins. Virology.

[CR75] Sidhu SS (2001). Engineering M13 for phage display. Biomol Eng.

[CR76] Sidhu SS, Fairbrother WJ, Deshayes K (2013). Exploring protein–protein interactions with phage display. ChemBioChem.

[CR77] Smith GP (1985). Filamentous fusion phage: novel expression vectors that display cloned antigens on the virion surface. Science.

[CR78] Smith GP (1993). Preface. Surface display and peptide libraries. Gene.

[CR79] Smith GP, Petrenko VA (1997). Phage display. Chem Rev.

[CR80] Smith J, Kontermann RE, Embleton J (2005). Antibody phage display technologies with special reference to angiogenesis. FASEB J.

[CR81] Soykut EA, Dudak FC, Boyaci IH (2008). Selection of staphylococcal enterotoxin B (SEB)-binding peptide using phage display technology. BiochemBiophys Res Commun.

[CR82] Stewart GS, Smith T, Denyer S (1989). Genetic engineering for bioluminescent bacteria. Food Sci Technol.

[CR83] Switała-Jelen K, Dąbrowska K, Górski A (2002). Mutations in bacteriophage T4 genome. Acta Virol.

[CR84] Ulitzur S, Kuhn J, Schlomerich J, Andreesen R, Kapp A (1987). Introduction of *lux* genes into bacteria, a new approach for specific determination of bacteria and their antibiotic susceptibility. Bioluminescence and chemiluminescence new perspectives.

[CR85] van Kessel JC, Hatfull GF (2008). Mycobacterial recombineering. Methods Mol Biol.

[CR86] Wang W, Chen X, Li T (2013). Screening a phage display library for a novel FGF8b-binding peptide with anti-tumor effect on prostate cancer. Exp Cell Res.

[CR87] Willats GT (2002). Phage display: practicalities and prospects. Plant Mol Biol.

[CR88] Yacoby I, Shamis M, Bar H (2006). Targeting antibacterial agents by using drug-carrying filamentous bacteriophages. Antimicrob Agents Chemother.

[CR89] Yacoby I, Bar H, Benhar I (2007). Targeted drug-carrying bacteriophages as antibacterial nanomedicines. Antimicrob Agents Chemother.

[CR90] Yang G, Cheng H, Liu C (2003). Inhibition of *Staphylococcus aureus* pathogenesis in vitro and in vivo by RAP-binding peptides. Peptides.

[CR91] Yoichi M, Abe M, Miyanaga K (2005). Alteration of tail fiber protein gp38 enables T2 phage to infect Escherichia coli O157:H7. J Biotechnol.

[CR92] Zoller MJ, Smith M (1982). Oligonucleotide-directed mutagenesis using M13-derived vectors: an efficient and general procedure for the production of point mutations in any fragment of DNA. Nucleic Acids Res.

[CR93] Zoller MJ, Smith M (1983). Oligonucleotide-directed mutagenesis of DNA fragments cloned into M13 vectors. Methods Enzymol.

